# Do patients and clinicians agree on the essential features of OCD?

**DOI:** 10.1017/S1092852926101011

**Published:** 2026-06-17

**Authors:** Maria Eduarda Moreira-de-Oliveira, Gabriela B. de Menezes, Leonardo F. Fontenelle

**Affiliations:** 1 Obsessive, Compulsive, and Anxiety Spectrum Research Program, Institute of Psychiatry, Federal University of Rio de Janeiro, Rio de Janeiro, Brazil; 2 D’Or Institute for Research and Education, Brazil; 3 Department of Psychiatry, Monash University, Clayton, Brazil

**Keywords:** Obsessive-compulsive disorder, phenomenological characteristics, DSM terminology

## Abstract

**Background:**

It is unclear whether patients with obsessive-compulsive disorder (OCD) and expert clinicians agree on the symptoms’ qualities that are essential for a diagnosis of OCD. This study examined whether OCD patients and expert clinicians agree on the essential characteristics for an OCD diagnosis. It compared the endorsement rates of various descriptive terms and assessed whether response profiles could predict group membership (patients versus expert clinicians).

**Methods:**

Sixty-four OCD patients and 36 OCD expert clinicians answered a questionnaire on their views regarding the defining characteristics of OCD symptoms. It included a compilation of adjectives extracted from DSM-5 to describe disorders in the compulsive–impulsive spectrum. Chi-square tests compared response prevalence between groups and binary regression identified distinguishing features. The significance level was set at 0.05.

**Results:**

All descriptors differed significantly between OCD patients and expert clinicians (*p* < 0.01), with clinicians consistently endorsing more items as applicable or essential. Although the logistic regression model significantly distinguished the 2 groups and showed high explanatory power (*R*^2^ = 0.7) with 99% classification accuracy, no individual descriptors were significant predictors.

**Conclusions:**

There is a mismatch between clinician-defined diagnostic language and patients’ subjective experience of OCD, including core symptom descriptors. This gap suggests that current OCD diagnostic construct may not fully capture how patients perceive their condition, potentially limiting its validity and clinical utility. Adopting more patient-centered language could improve assessment, psychoeducation, and treatment engagement.

## Introduction

Obsessive-compulsive disorder (OCD) is a debilitating psychiatric condition with a global lifetime prevalence of approximately 3%.^1^ It profoundly affects quality of life, occupational functioning, and interpersonal relationships.^2^^,^^3^ Individuals with OCD are more likely to experience unemployment, lower socioeconomic status, strained social and marital relationships, and increased healthcare utilization.^3^ Consequently, OCD imposes substantial and enduring economic burdens, including direct costs associated with treatment and healthcare services, as well as indirect costs related to reduced personal income, employment, and national economic productivity.^4^

Unfortunately, the disorder often goes unrecognized in primary care and psychiatric settings, partly due to the internal and less observable nature of many symptoms.^5^ Furthermore, in the absence of highly specific biological markers, diagnosis and research continue to depend heavily on the evaluation of clinical symptoms. It is crucial to align the perspectives of patients and healthcare providers, ensuring they communicate using a shared, accurate “language” regarding the disorder. For instance, OCD patients, as the ones experiencing symptoms, may interpret their condition through the lens of personal struggle. In contrast, clinicians might prioritize the functional or external manifestations of symptoms, favoring more objective (or observable) characteristics.

A recent qualitative study on mental imagery in OCD investigated individuals’ experiences through interviews, identifying common characteristics of OCD-related imagery.^5^ Terms such as “unpleasant” and “intrusive” were frequently used to describe patients’ experiences, but other descriptors, including “intense,” “overwhelming,” “uncontrollable,” “spontaneous,” “influenced,” “irrational,” and “illogical,” also emerged. These adjectives highlight the complexity and multifaceted nature of OCD experiences. Yet, a deeper understanding regarding their meaning and use could bridge the gap between subjective experiences and clinical descriptions, providing insights that are directly relevant to both clinical practice and research.^6^^,^^7^

In the present paper, we investigated whether OCD patients and expert clinicians agree about the qualities of the diagnostic criteria (eg, thoughts, urges, images, behaviors) deemed important (or essential) for a diagnosis of OCD. To do so, we compared OCD patients’ self-reported descriptions of their own symptom experience with independent expert clinicians’ professional conceptualizations of OCD, based on the rates of endorsement of a series of descriptive adjectives. These terms were sourced from DSM-5 criteria for disorders characterized by compulsive (ie, obsessive-compulsive and related disorders), impulsive (ie, eating, disruptive, impulse-control, and conduct disorders), or addictive (ie, substance-related and addictive disorders) features. They were chosen because they were thought to represent qualities common to experiences within the so-called compulsive–impulsive spectrum.^8^

We predicted it would be possible to differentiate OCD patients and expert clinicians based on the qualities thought to be important for obsessive-compulsive symptoms, with OCD patients using more subjective (or less observable) adjectives to describe their symptoms (eg, intrusive, unwanted) and expert clinicians using more objective (or more observable) adjectives (eg, repetitive, inflexible) to characterize their OCD patients’ symptoms. In contrast, inability to different OCD patients and expert clinicians in regression models would indicate that they generally agree in terms of the adjectives used to characterize OCD symptoms.

## Methods

### Participants

The sample comprised 64 patients with OCD and 36 expert clinicians specializing in OCD from various Brazilian mental health centers. Patients were consecutively recruited from the OCD Clinic, part of the Obsessive, Compulsive, and Anxiety Spectrum Research Program at the Institute of Psychiatry, Federal University of Rio de Janeiro. A total of 71 patients were approached for participation, of whom 64 consented and completed the study procedures. OCD diagnoses were confirmed using the Mini International Neuropsychiatric Interview (MINI).^9^ Inclusion criteria for patients were: (i) a primary diagnosis of OCD; (ii) age ≥ 18 years; and (iii) the ability to read and complete forms.

Expert clinicians were eligible if they were psychiatrists or psychologists with a minimum of 2 self-disclosed years of clinical experience in the diagnosis and treatment of OCD. This experience must have been acquired in specialized clinics within private or public institutions that offer dedicated services for patients with OCD across the country. A total of 35 psychiatrists and 30 psychologists were invited to participate, of whom 36 clinicians completed the questionnaire. Of these, 22 were psychiatrists and 14 were psychologists. Recruitment of expert clinicians occurred through in-person invitations or email outreach.

The study protocol was approved by the local ethics committee, and all participants provided written informed consent. Initial patient assessments were conducted in person at the clinic (September 2018–January 2020). Due to the COVID-19 pandemic, subsequent data collection transitioned to a remote format, concluding in October 2020. Remote data collection utilized the Research Electronic Data Capture (REDCap) platform hosted by the D’Or Institute for Research and Education. All data, except for the MINI assessment - administered by a specialist either in person or via videoconference during the pandemic - were obtained through self-reported instruments. Therefore, the transition from in-person to remote assessments did not impact the measurement process. Expert clinician assessments began in person during a national psychiatric conference (October 2018), with subsequent data collected remotely via REDCap from June 2019 to January 2021.

### Measures

#### Demographics

Patients responded to a questionnaire that included information on age, gender, education, ethnicity, marital status, and employment status. Expert clinicians responded to a similar questionnaire that included information on age, gender, years of practice in general, and years of practice with OCD patients.

#### OCD-related terminology questionnaire

This paper employed 2 comparable versions of a newly built questionnaire to assess common experiences of individuals with OCD: one designed for patients and another for expert clinicians. Both versions were divided into 4 sections (Portuguese and English versions are provided in the Supplementary Material). For the purposes of this study, analyses were restricted to data from Section 1. This analytic focus was determined by the authors and was not prespecified prior to data collection; the remaining sections of the questionnaire are reserved for future analyses and publications.

The first section presented a list of adjectives extracted from the DSM-5 to describe signs and symptoms associated with *Obsessive-Compulsive and Related Disorders*; *Substance-Related and Addictive Disorders*; *Disruptive, Impulse-Control, and Conduct Disorders*; and *Feeding and Eating Disorders*. Examples of included terms were “recurrent,” “persistent,” and “intrusive.” Patients were instructed to select all words they believe accurately describe their symptoms, whereas expert clinicians classified each term as not applicable, applicable, or essential for describing OCD symptoms. For analysis, terms classified as applicable were combined with those categorized as essential to diagnosis. Comparisons restricted to essential ratings versus patient classifications are presented in Supplementary Material (Supplementary Table 1).

The second section included objects, themes, or situations that individuals attempted to avoid or restrict due to signs and symptoms, such as contaminated objects, mirrors or reflective surfaces, or the act of discarding possessions or objects. Sections 3 and 4 shared the same structure and wording, differing only in focus: behaviors versus “mental experiences” (eg, cognitions). These sections explored perceptions of the purpose, consequences, and antecedents of behaviors or and mental experiences.

#### Symptoms’ severity (for OCD patients)

To assess severity of OCD symptoms, the self-report Yale-Brown Obsessive-Compulsive Scale (Y-BOCS) was administered. The Y-BOCS is the most widely used instrument to measure the severity of OCD. It includes a total of 10 items that cover time, interference, anxiety or distress, resistance, and control for obsessions and compulsions separately.^10^^,^^11^ At the same time, the scores on different symptom dimensions were evaluated through the OCI-R,^12^^,^^13^ an 18-item self-report scale assessing 6 areas of obsessive-compulsive experiences over the month before. Patients also completed the Depression Anxiety Stress Scale (DASS).^14^^,^^15^ The DASS-21 contains 21 self-report items assessing depression, anxiety, and stress/tension symptoms. The measurement of interest in this study was the total score, reflecting general psychological distress.

### Data analysis

Descriptive statistics were described in percentages, means, and standard deviations. The prevalence of each potential OCD phenomenological feature was compared between groups using chi-square tests. Binary logistic regression was then conducted to identify which features significantly differentiated group membership (patients versus expert clinicians). The single missing value observed in the clinician group was handled by casewise exclusion in the relevant analyses. Significance was set at .05. The analyses were carried out through SPSS version 23.0.

## Results

### Descriptive statistics

A detailed summary of the sociodemographic and clinical characteristics is presented in Table 1. Clinically, the patient sample exhibited moderate OCD symptom severity, with a mean Y-BOCS score of 21.6 (SD = 8). OCI-R subscale scores were as follows (mean ± SD): washing = 4.35 ± 4.06, checking = 4.05 ± 3.75, ordering = 3.29 ± 3.74, obsessing = 6.87 ± 3.19, hoarding = 2.47 ± 3.34, and neutralizing = 3.15 ± 3.70. Psychological distress, as measured by the DASS-21, showed a mean score of 22.93 (*SD* = 16.10).

**Table 1.** Sample demographics and clinical variables Table 1. long description.

Patients		
Age, mean (SD)		40.8 (13.9)
Gender, *N* (%)		
Female		34 (53.1)
Marital status, *N* (%)		
Single		41 (64.1)
Married		17 (26.6)
Divorced		5 (7.8)
Widowed		1 (1.6)
Ethnicity, *N* (%)		
Caucasian		39 (60.9)
Black		7 (10.9)
Asian		2 (3.1)
Other		16 (25.0)
Education, *N* (%)		
Up to 8 years		1 (1.6)
9–11 years		8 (12.5)
12 years or more		54 (84.4)
Missing		1 (1.6)
Occupation, *N* (%)		
Working		25 (39.1)
Unemployed		14 (21.9)
Retired		10 (14.9)
Student		5 (7.8)
Other		9 (14.0)
Missing		1 (1.6)
Duration of illness, mean (SD)		23.73 (13.8)
Comorbidities, *N* (%)		
MD		20 (31.3)
PD		3 (4.7)
Agoraphobia		13 (20.3)
SAD		9 (14.1)
PTSD		3 (4.7)
GAD		17 (26.6)

Clinicians		
Age, mean (SD)		44.6 (12.3)
Gender, *N* (%)		
Female		25 (69.4)
Specialty, *N* (%)		
Psychiatry		22 (61.1)
Psychology		14 (38.9)
Years of practice, mean (SD)		18.8 (12.2)
Years of practice with OCD, mean (SD)		15.8 (9.5)

Abbreviations: MD, major depression; PD, panic disorder; SAD, social anxiety disorder; PTSD, post-traumatic stress disorder; GAD, generalized anxiety disorder.

### OCD symptoms’ qualities

Each quality of OCD symptoms, as represented by adjectives extracted from DSM-5, was compared between OCD patients and expert clinicians (Table 2). Significant differences were observed between patients and clinicians across all phenomenological descriptors (all *p* < 0.01). Clinicians consistently endorsed a higher proportion of items as applicable or essential compared to patients. Notably, even core DSM descriptors such as “recurrent,” “intrusive,” and “persistent” were not universally endorsed by patients, whereas clinicians almost uniformly identified them as relevant.

**Table 2.** OCD symptoms definition Table 2. long description.

	DSM-5 chapter source	Expert clinicians		OCD patients		Statistics	General findings
		[N_yes_ (%)]	[N_missing_ (%)]	[N_yes_ (%)]	[N_missing_ (%)]		
Recurrent	**OCD**, TR, SP, BN, BED, IED, KM, AUD, CUD, PUD, OHD, IUD, OUD, SHA, SUD, TUD, OSD, GD	35 (97.2)	0 (0.0)	27 (42.2)	0 (0.0)	p < 0.001*	Clinician > Patient
Persistent	**OCD**, HD, AN, CD, AUD, CUD, PUD, OHD, IUD, OUD, SHA, SUD, TUD, OSD, GD	36 (100.0)	0 (0.0)	36 (56.3)	0 (0.0)	p < 0.001*	Clinician > Patient
Intrusive	**OCD**, BDD	36 (100.0)	0 (0.0)	32 (50.0)	0 (0.0)	p < 0.001*	Clinician > Patient
Unwanted	**OCD**, BDD	36 (100.0)	0 (0.0)	49 (76.6)	0 (0.0)	p = 0.002*	Clinician > Patient
Repetitive	**OCD**, BDD, CD	36 (100.0)	0 (0.0)	46 (71.9)	0 (0.0)	p < 0.001*	Clinician > Patient
Excessive	**OCD**, BDD, HD, BN, BED	34 (97.1)	1 (2.8)	24 (37.5)	0 (0.0)	p < 0.001*	Clinician > Patient
Inappropriate	BN	35 (97.2)	0 (0.0)	23 (35.9)	0 (0.0)	p < 0.001*	Clinician > Patient
Continued	AUD, CUD, PUD, OHD, IUD, OUD, SHA, SUD, TUD, OSD	29 (82.9)	1 (2.8)	24 (37.5)	0 (0.0)	p < 0.001*	Clinician > Patient
Unpleasant	AUD	34 (97.1)	1 (2.8)	48 (75.0)	0 (0.0)	p = 0.005*	Clinician > Patient
Habitual	AUD	31 (88.6)	1 (2.8)	18 (28.1)	0 (0.0)	p < 0.001*	Clinician > Patient
Inflexible	AN	34 (97.1)	1 (2.8)	8 (12.5)	0 (0.0)	p < 0.001*	Clinician > Patient
Unproductive	–	31 (86.1)	0 (0.0)	22 (34.4)	0 (0.0)	p < 0.001*	Clinician > Patient
Compensatory	BN	29 (82.9)	1 (2.8)	7 (10.9)	0 (0.0)	p < 0.001*	Clinician > Patient

Abbreviations: OCD, obsessive-compulsive disorder; BDD, body dysmorphic disorder; HD, hoarding disorder; TR, trichotillomania; SP, skin-picking; AN, anorexia nervosa; BN, bulimia nervosa; BED, binge-eating disorder; IED, intermittent explosive disorder; CD, conduct disorder; PM, pyromania; KM, kleptomania; AUD, alcohol use disorder; CUD, cannabis use disorder; PUD, phencyclidine use disorder; OHD, other hallucinogen use disorder; IUD, inhalant use disorder; OUD, opioid use disorder; SHA, sedative, hypnotic, or anxiolytic use disorder; SUD, stimulant use disorder; TUD, tobacco use disorder; OSD, other (or unknown) substance use disorder; GD, gambling disorder; *, *p* < 0.05.

A binary logistic regression analysis was conducted to examine whether DSM-derived phenomenological descriptors distinguish OCD patients from clinicians. The overall model was significant, χ^2^(13) = 118.13, *p* < 0.001, explaining a large proportion of variance (Cox and Snell *R*^2^ = 0.7) and correctly classifying 99% of cases. However, none of the individual predictors reached statistical significance in the final model, and coefficient estimates were unstable, indicating quasi-complete separation.

## Discussion

Our findings indicate a discrepancy between how OCD patients define their condition and what expert clinicians consider important or essential for a diagnosis of OCD, raising concerns about both the validity and the communicability of current diagnostic language. Clinicians were substantially more likely than patients to endorse the researched phenomenological features as applicable or essential (as opposed to not applicable). This difference was also noted in relation to peripheral or ambiguous descriptors that are not described in DSM OCD chapter.

The largest discrepancies were observed for terms such as “inflexible” and “compensatory” - originally derived from the DSM-5 criteria for bulimia nervosa (BN) and anorexia nervosa (AN).^16^ Despite 97.1% and 82.9% of expert clinicians identifying “inflexibility” and a “compensatory” nature of symptoms as relevant for OCD, these traits were not emphasized by OCD patients as important features of their disorder. While research suggests that OCD and eating disorders share some clinical and biological similarities,^17^^,^^18^ our data indicate that OCD patients do not view these characteristics as central to their condition, even though they play a critical role in the diagnosis of eating disorders.

In addition, logistic regression analysis indicated that, although the overall pattern of responses clearly differentiated patients from clinicians, no individual descriptor emerged as a stable predictor. The lack of significant independent effects, combined with instability in the estimates, suggests that these variables function as a highly intercorrelated set rather than as discrete features. This pattern indicates that the phenomenological language used in diagnostic systems may not fully correspond to the answers given in the questionnaire, which are thought to reflect the patients’ “lived experience”.

Our findings have important clinical implications. If patients do not spontaneously recognize core diagnostic descriptors as representative of their symptoms, this misalignment may compromise the accuracy of assessment, as self-report and clinician-based evaluations may rely on partially divergent conceptual frameworks. Limited correspondence between diagnostic terminology and subjective experience may also reduce the effectiveness of psychoeducation, impairing patients’ understanding and acceptance of their condition. This, in turn, may hinder treatment engagement, given that the perceived relevance and credibility of clinical formulations are central to adherence and therapeutic alliance. Future research should qualitatively examine how patients perceive and articulate their symptoms to better align diagnostic language with terms that are more patient-centric and accessible.

Despite its insights, our study has some limitations. The first concerns sample size. Although we recruited a substantial cohort of OCD patients from a specialized clinic, larger samples may capture a broader range of symptoms and perspectives on OCD diagnosis. In addition, both patient and clinician samples were recruited using convenience-based procedures and largely drawn from a single clinical center or professional network, which may limit the generalizability of the findings and favor the participation of individuals who are more motivated, better educated, or more familiar with OCD-related concepts.

Second, because all participants were primarily Brazilian Portuguese-speaking, cultural factors - including the nuanced meanings of certain adjectives (eg, “inappropriate”) - may have influenced our findings. Although the questionnaire was based on wording extracted directly from the DSM-5 and did not require translation for data collection, the instrument itself has not undergone formal psychometric validation, and conclusions should therefore be considered exploratory and contingent on the use of a non-validated measure.

Thirdly, although “compensatory” and “inflexible” may seem too technical for OCD patients to use, all adjectives were sourced from the same reference - the DSM-5 - which probably adopts a similar level of technical language to describe different disorders. Nevertheless, the selection and framing of adjectives, as well as the closed-ended structure of the questionnaire, may have shaped response patterns and contributed to observed group differences.

Additionally, the phrasing of some survey questions (eg, “My problem can be defined as…”) could have been interpreted either as a personal description of the patient’s own experience or as a general characterization of OCD. Consequently, some patients may have described OCD as “repetitive” not necessarily because they perceive their own symptoms that way, but because they have encountered this description in countless educational materials or specialized websites.

In this regard, it is also important to note a structural asymmetry in the study design: patients were asked to describe their own lived experience, whereas clinicians were asked to characterize OCD from a professional perspective, and clinicians may have adopted different frames of reference (eg, typical patients, their own clinical caseload, or OCD as a diagnostic entity), contributing to variability in responses. Moreover, differences in response formats between patient and clinician versions, including the use of graded importance ratings for clinicians, further limit the strength of inferences regarding agreement or disagreement on what constitutes “essential” features of OCD.

Furthermore, patient clinical characterization was limited in scope, as information on treatment status was not systematically collected, and unmeasured clinical factors - such as treatment exposure or illness stage - may have influenced how symptoms were conceptualized and verbalized. Another limitation is that the clinician group likely encompassed varying levels and types of OCD-related expertise, as eligibility criteria were based on minimal, self-reported experience, and more detailed information on training, clinical setting, or caseload was not available.

Data collection occurred both in person and online and spanned pre- and intra-pandemic periods; although the questionnaire consisted of structured, closed-ended items with identical content across formats, potential effects of administration mode and broader contextual influences cannot be fully excluded. Finally, the exploratory analytic approach, including multiple uncorrected comparisons and regression modeling in a modest sample, raises the possibility of overfitting and further supports a cautious interpretation of the findings. Nevertheless, despite these limitations, our study may represent the first attempt to understand, in a more quantitative way, the levels of agreement regarding the subjective experiences of OCD in patients versus people who assess and treat them.

## Supporting information

10.1017/S1092852926101011.sm001 Moreira-de-Oliveira et al. supplementary material 1 Moreira-de-Oliveira et al. supplementary material

10.1017/S1092852926101011.sm002 Moreira-de-Oliveira et al. supplementary material 2 Moreira-de-Oliveira et al. supplementary material

## Supplementary material

The supplementary material for this article can be found at http://doi.org/10.1017/S1092852926101011.

## Long descriptions

### Table 1. Long description

The table is divided into two primary sections: Patients and Clinicians.

Patient Demographics (N = 64):

* Age: mean 40.8, S D 13.9.

* Gender: Female 34 (53.1 percent).

* Marital status: Single 41 (64.1 percent), Married 17 (26.6 percent), Divorced 5 (7.8 percent), Widowed 1 (1.6 percent).

* Ethnicity: Caucasian 39 (60.9 percent), Black 7 (10.9 percent), Asian 2 (3.1 percent), Other 16 (25.0 percent).

* Education: Up to 8 years 1 (1.6 percent), 9 to 11 years 8 (12.5 percent), 12 years or more 54 (84.4 percent), Missing 1 (1.6 percent).

* Occupation: Working 25 (39.1 percent), Unemployed 14 (21.9 percent), Retired 10 (14.9 percent), Student 5 (7.8 percent), Other 9 (14.0 percent), Missing 1 (1.6 percent).

* Duration of illness: mean 23.73, S D 13.8.

* Comorbidities: M D 20 (31.3 percent), P D 3 (4.7 percent), Agoraphobia 13 (20.3 percent), S A D 9 (14.1 percent), P T S D 3 (4.7 percent), G A D 17 (26.6 percent).

Clinician Demographics (N = 36):

* Age: mean 44.6, S D 12.3.

* Gender: Female 25 (69.4 percent).

* Specialty: Psychiatry 22 (61.1 percent), Psychology 14 (38.9 percent).

* Years of practice: mean 18.8, S D 12.2.

* Years of practice with O C D: mean 15.8, S D 9.5.

Abbreviations: M D (major depression), P D (panic disorder), S A D (social anxiety disorder), P T S D (post-traumatic stress disorder), G A D (generalized anxiety disorder).

Navigate back to Table 1..

### Table 2. Long description

The table consists of eight columns: Symptom term, D S M-5 chapter source, Expert clinicians N yes percentage, Expert clinicians N missing percentage, O C D patients N yes percentage, O C D patients N missing percentage, Statistics, and General findings.

Key data rows include:

* Recurrent: Clinicians 97.2 percent vs Patients 42.2 percent. p is less than 0.001.

* Persistent: Clinicians 100.0 percent vs Patients 56.3 percent. p is less than 0.001.

* Intrusive: Clinicians 100.0 percent vs Patients 50.0 percent. p is less than 0.001.

* Unwanted: Clinicians 100.0 percent vs Patients 76.6 percent. p equals 0.002.

* Repetitive: Clinicians 100.0 percent vs Patients 71.9 percent. p is less than 0.001.

* Excessive: Clinicians 97.1 percent vs Patients 37.5 percent. p is less than 0.001.

* Inappropriate: Clinicians 97.2 percent vs Patients 35.9 percent. p is less than 0.001.

* Continued: Clinicians 82.9 percent vs Patients 37.5 percent. p is less than 0.001.

* Unpleasant: Clinicians 97.1 percent vs Patients 75.0 percent. p equals 0.005.

* Habitual: Clinicians 88.6 percent vs Patients 28.1 percent. p is less than 0.001.

* Inflexible: Clinicians 97.1 percent vs Patients 12.5 percent. p is less than 0.001.

* Unproductive: Clinicians 86.1 percent vs Patients 34.4 percent. p is less than 0.001.

* Compensatory: Clinicians 82.9 percent vs Patients 10.9 percent. p is less than 0.001.

In all categories, the general finding is Clinician greater than Patient, indicating clinicians endorse these descriptive terms for O C D symptoms at significantly higher rates than patients do.

Navigate back to Table 2..
