# Supplementary material for: Do patients and clinicians agree on the essential features of OCD?
Source: CNS Spectr. 2026 Jun 17;31(1):e21. doi: 10.1017/S1092852926101011 (PMC13732608; doi:10.1017/S1092852926101011)
Supplement: Supplementary file 1 [file S1092852926101011sup001.pdf]

OCD - CLINICIAN QUESTIONNAIRE

Identification: ________________________________ **Date: ___/___/_____**

VALIDITY OF DSM-5 LINGUISTIC DESCRIPTIONS FOR OCD (VDLD-OCD)

In this questionnaire, we will be asking questions about obsessive-compulsive disorder ***behaviors*** (e.g. compulsive behaviors) and obsessive-compulsive ***mental experiences*** (e.g. obsessional thoughts). To answer all the following questions, please consider *only* your *personal opinion* based on your clinical experience.

SECTION 1

Please read the terms below used by DSM-5 to describe several symptoms that are typical of different mental disorders and rate the extent to which they apply to define obsessive-compulsive symptoms (either behaviors and/or mental experiences):

0 = Do not apply **1 = Can apply** **2 = Apply (essential to diagnosis)**

☐ Recurrent	☐ Excessive	☐ Inflexible
☐ Persistent	☐ Inappropriate	☐ Unproductive
☐ Intrusive	☐ Continued	☐ Compensatory
☐ Unwanted	☐ Unpleasant	
☐ Repetitive	☐ Habitual	

SECTION 2

IN YOUR OPINION, OBSESSIVE-COMPULSIVE SYMPTOMS ARE *TIPICALLY* CHARACTERIZED BY AN ATTEMPT TO AVOID OR RESTRICT: [tick one or more options]

( ) Contaminated objects
( ) Risky situations (unrelated to contamination)
( ) Sexual or blasphemous themes
( ) Asymmetric environments
( ) Mirror or reflective surfaces
( ) Discarding possessions or objects
( ) Energy intake or food
( ) Social situations
( ) Public transportation
( ) Open spaces
( ) Enclosed spaces
( ) Standing in line
( ) Crowds
( ) Leaving home
( ) Other objects or situations
( ) They are not an attempt to avoid or restrict anything

SECTION 3

➢ To answer the following questions, please refer ONLY to obsessive-compulsive ***behaviors***.

OCD PATIENTS' *BEHAVIORS* INCLUDE: [tick one or more options]

( ) Hand washing
( ) Ordering
( ) Checking
( ) Mirror checking
( ) Excessive grooming
( ) Not discarding possessions
( ) Acquisition
( ) Skin picking
( ) Reassurance seeking
( ) Pulling out of one's hair
( ) Eating (Energy intake)
( ) Outbursts (aggressive impulses)
( ) Fire setting
( ) Stealing
( ) Using alcohol
( ) Using drugs
( ) Gambling
( ) Praying
( ) Counting
( ) Repeating words silently
( ) Comparing appearance with that of others
( ) Other behavior
( ) They are not behaviors

If you chose one or more behaviors, go to letter A.
If you chose the option "They are not behaviors", go to Section 4.

A) I THINK THAT BEHAVIORS OF PATIENTS WITH OCD ARE: [tick one or more options]

I. ( ) Clearly excessive
II. ( ) Associated with lack of control
III. ( ) Associated with repeated and unsuccessful attempts to be:

a. ( ) Decreased or cut down
b. ( ) Stopped
c. ( ) Ignored
d. ( ) Suppressed
e. ( ) Neutralized
f. ( ) Resisted

IV. ( ) Designed to neutralize or prevent other phenomena:

a. ( ) that they ***are*** realistically connected with (i.e. behaviors of OCD patients are logically related to what they aim to neutralize/prevent)
b. ( ) that they ***are not*** realistically connected with (i.e. behaviors of OCD patients are not logically related to what they aim to neutralize/prevent)

V. ( ) None of the above

Now keep answering the following questions.

B) OCD PATIENTS' BEHAVIORS ARE PERFORMED DESPITE: [tick one or more options]

( ) Social or interpersonal problems
( ) Physical hazards
( ) Psychological problems
( ) None of the above

C) SOME OF THE OCD PATIENTS' BEHAVIORS LEAD TO: [tick one or more options]

( ) Anxiety or distress
( ) Pleasure or gratification
( ) Relief
( ) None of the above

D) WHILE OTHERS ARE AIMED AT: [tick one or more options]

( ) Preventing anxiety or distress
( ) Reducing anxiety or distress
( ) Preventing some dreaded event or situation
( ) Obtaining pleasure or gratification
( ) Obtaining relief
( ) None of the above

E) OCD PATIENTS' <u>BEHAVIORS</u> ARE PERFORMED IN RESPONSE OR ACCORDING TO: [tick one or more options]

( ) Thoughts
( ) Urges
( ) Images
( ) Preoccupations
( ) Fascinations or attractions
( ) Interests or curiosities
( ) Desires or cravings
( ) Needs
( ) Feelings
( ) Fears
( ) Appearance concerns
( ) Rigid rules
( ) A force
( ) An obligation
( ) A pressure
( ) None of the above

F) OCD PATIENTS' <u>BEHAVIORS</u> ARE PRECEEDED BY: [tick one or more options]

( ) Tension
( ) Affective arousal
( ) None of the above

G) OCD PATIENTS' <u>BEHAVIORS</u> OCCUR WHEN THE PATIENT IS FEELING: [tick one or more options]

( ) Anxious
( ) Depressed
( ) Helpless
( ) Guilty
( ) None of the above

H) OCD PATIENTS' <u>BEHAVIORS</u> LEAD THEM TO: [tick one or more options]

( ) Need increased amounts of behavior to achieve the desired effect
( ) Become restless or irritable when attempting to cut down or stop performing behavior
( ) Unsuccessfully attempt to control, cutback or stop the behavior
( ) Become preoccupied with the behavior
( ) Perform the behavior when feeling distressed (e.g. helpless, guilty, anxious, depressed)
( ) Return to get things even
( ) Lie to conceal the extent of involvement with behaviors
( ) Jeopardize or lose a significant relationship, job, or educational career opportunity because of behavior
( ) Rely on others because of behaviors
( ) None of the above

Go to <u>Section 4</u>.

SECTION 4

- To answer the following questions, please refer ONLY to obsessive-compulsive ***mental experiences*** (e.g. cognitions).

OCD PATIENTS' *MENTAL EXPERIENCES* INCLUDE: [tick one or more options]

( ) Thoughts
( ) Urges
( ) Images
( ) Preoccupations
( ) Fascinations or attractions
( ) Interests or curiosities
( ) Desires or cravings
( ) Needs
( ) Feelings
( ) Fears
( ) Other mental experience
( ) They are not mental experiences

If you chose one or more mental experiences, go to letter A.
If you chose the option "They are not mental experiences", stop answering this questionnaire.

A) I THINK THAT MENTAL EXPERIENCES OF PATIENTS WITH OCD ARE: [tick one or more options]

I. ( ) Clearly excessive
II. ( ) Associated with lack of control
III. ( ) Associated with repeated and unsuccessful attempts to be:

a. ( ) Decreased or cut down
b. ( ) Stopped
c. ( ) Ignored
d. ( ) Suppressed
e. ( ) Neutralized
f. ( ) Resisted

IV. ( ) Designed to neutralize or prevent other phenomena:

g. ( ) that they ***are*** realistically connected with (i.e. mental experiences of OCD patients are logically related to what they aim to neutralize/prevent)
h. ( ) that they ***are not*** realistically connected with (i.e. mental experiences of OCD patients are not logically related to what they aim to neutralize/prevent)

V. ( ) None of the above

Now keep answering the following questions.

B) OCD PATIENTS' MENTAL EXPERIENCES HAPPEN DESPITE: [tick one or more options]

( ) Social or interpersonal problems
( ) Physical hazards
( ) Psychological problems
( ) None of the above

C) SOME OF OCD PATIENTS' <u>MENTAL EXPERIENCES</u> LEAD TO: [tick one or more options]

( ) Anxiety or distress
( ) Pleasure or gratification
( ) Relief
( ) None of the above

D) WHILE OTHERS ARE AIMED AT: [tick one or more options]

( ) Prevent anxiety or distress
( ) Reduce anxiety or distress
( ) Prevent some dreaded event or situation
( ) Get him/her pleasure or gratification
( ) Get him/her relief
( ) None of the above

E) OCD PATIENTS' <u>MENTAL EXPERIENCES</u> ARE PRECEEDED BY: [tick one or more options]

( ) Tension
( ) Affective arousal
( ) None of the above

F) OCD PATIENTS' <u>MENTAL EXPERIENCES</u> OCCUR WHEN THE PATIENT IS FEELING: [tick one or more options]

( ) Anxious
( ) Depressed
( ) Helpless
( ) Guilty
( ) None of the above

G) OCD PATIENTS ALSO: [tick one or more options]

( ) Need increased amounts of the mental experience to feel satisfied
( ) Become restless or irritable when attempting to not have the mental experience
( ) Feel unable to control, cutback or stop the mental experience
( ) Become preoccupied with the mental experience
( ) Experience it when feeling distressed (e.g. helpless, guilty, anxious, depressed)
( ) Lie to conceal the mental experience
( ) Jeopardize or lose a significant relationship, job, or educational career opportunity because of the mental experience
( ) Rely on others because of the mental experience
( ) None of the above

TOC – QUESTIONÁRIO CLÍNICO

Identificação: ___________________________________ **Data: ___/___/_____**

VALIDAÇÃO DAS DESCRIÇÕES LINGUÍSTICAS DO DSM-5 PARA O TOC (VDLD-TOC)

Nesse questionário faremos perguntas sobre ***comportamentos*** (comportamentos compulsivos) e ***experiências mentais*** (pensamentos obsessivos) do transtorno obsessivo-compulsivo (TOC). Para responder às seguintes questões, favor considerar *apenas* sua *opinião pessoal* baseada em sua experiência clínica.

SEÇÃO 1

Por favor, leia os termos abaixo utilizados pelo DSM-5 para descrever uma série de sintomas característicos de diferentes transtornos mentais e indique o quanto eles se aplicam à definição de sintomas obsessivo-compulsivos (incluindo comportamentos e/ou experiências mentais):

0 = Não se aplica	1 = Pode aplicar-se	2 = Aplica-se (essencial para o diagnóstico)

- ☐ Recorrentes
- ☐ Persistentes
- ☐ Intrusivos
- ☐ Indesejados
- ☐ Repetitivos
- ☐ Excessivos
- ☐ Inapropriados
- ☐ Contínuos
- ☐ Desagradáveis
- ☐ Habituais
- ☐ Inflexíveis
- ☐ Improdutivos
- ☐ Compensatórios

SEÇÃO 2

NA SUA OPINIÃO, SINTOMAS OBSESSIVO-COMPULSIVOS CARACTERIZAM-SE *TIPICAMENTE* POR TENTATIVAS DE EVITAR OU RESTRINGIR: [assinale uma ou mais opções]

( ) Objetos contaminados
( ) Situações de risco (não relacionadas à contaminação)
( ) Blasfêmias ou temas sexuais
( ) Ambientes assimétricos
( ) Espelhos ou superfícies refletoras
( ) Descartar pertences ou objetos
( ) Ingestão calórica ou alimentos
( ) Situações sociais
( ) Transporte público
( ) Espaços abertos
( ) Locais fechados
( ) Ficar em fila
( ) Multidões
( ) Sair de casa
( ) Outros objetos ou situações
( ) Não são uma tentativa de evitar ou restringir coisas

SEÇÃO 3

➢ Para responder às próximas questões, por favor refira-se APENAS a ***comportamentos*** obsessivo-compulsivos.

COMPORTAMENTOS DE PACIENTES COM TOC INCLUEM: [assinale uma ou mais opções]

( ) Lavagem de mãos
( ) Ordenamento
( ) Verificação/checagem
( ) Verificação no espelho
( ) Arrumação excessiva
( ) Não descartar pertences
( ) Aquisição
( ) Beliscar a pele
( ) Buscar tranquilização
( ) Arrancar os próprios cabelos
( ) Comer (ingestão calórica)
( ) Explosividade (impulsos agressivos)
( ) Provocar incêndios
( ) Roubar
( ) Uso de álcool
( ) Uso de drogas
( ) Apostas
( ) Rezas
( ) Contagem
( ) Repetição de palavras em silêncio
( ) Comparação da aparência com a de outros
( ) Outro comportamento
( ) Não são comportamentos

Se você escolheu um ou mais comportamentos, vá para a letra A.
Se você escolheu a opção "Não são comportamentos", vá para a Seção 4.

A) EU ACHO QUE COMPORTAMENTOS DE PACIENTES COM TOC: [assinale uma ou mais opções]

I. ( ) São claramente excessivos
II. ( ) Estão associados à falta de controle
III. ( ) Estão associados a tentativas repetidas e sem sucesso de:

  a. ( ) Reduzi-los
  b. ( ) Pará-los
  c. ( ) Ignorá-los
  d. ( ) Suprimi-los
  e. ( ) Neutralizá-los
  f. ( ) Resisti-los

IV. ( ) São projetados para neutralizar ou prevenir outro fenômeno:

  a. ( ) ***que está*** realisticamente conectado com eles (comportamentos de pacientes com TOC estão logicamente relacionados ao que eles visam neutralizar/prevenir)
  b. ( ) ***que não está*** realisticamente conectado com eles (comportamentos de pacientes com TOC não estão logicamente relacionados ao que eles visam neutralizar/prevenir)

V. ( ) Nenhuma das anteriores

Continue respondendo às questões seguintes.

B) COMPORTAMENTOS DE PACIENTES COM TOC SÃO DESEMPENHADOS, APESAR DE: [assinale uma ou mais opções]

( ) Problemas sociais ou interpessoais
( ) Riscos à integridade física
( ) Problemas psicológicos
( ) Nenhuma das anteriores

C) ALGUNS DOS COMPORTAMENTOS DE PACIENTES COM TOC LEVAM A: [assinale uma ou mais opções]

( ) Ansiedade ou sofrimento
( ) Prazer ou gratificação
( ) Alívio
( ) Nenhuma das anteriores

D) ENQUANTO OUTROS TÊM O OBJETIVO DE: [assinale uma ou mais opções]

( ) Prevenir ansiedade ou sofrimento
( ) Reduzir ansiedade ou sofrimento
( ) Prevenir algum evento ou situação temida
( ) Oferecer prazer ou gratificação
( ) Oferecer alívio
( ) Nenhuma das anteriores

E) <u>COMPORTAMENTOS</u> DE PACIENTES COM TOC SÃO DESEMPENHADOS EM REPOSTA OU DE ACORDO COM: [assinale uma ou mais opções]

( ) Pensamentos
( ) Impulsos
( ) Imagens
( ) Preocupações
( ) Fascinações ou atrações
( ) Interesses ou curiosidades
( ) Desejos ou fissuras
( ) Necessidades
( ) Sentimentos
( ) Medos
( ) Preocupações com a aparência
( ) Regras rígidas
( ) Uma força
( ) Uma obrigação
( ) Uma pressão
( ) Nenhuma das anteriores

F) <u>COMPORTAMENTOS</u> DE PACIENTES COM TOC SÃO PRECEDIDOS POR: [assinale uma ou mais opções]

( ) Tensão
( ) Excitação
( ) Nenhuma das anteriores

G) <u>COMPORTAMENTOS</u> DE PACIENTES COM TOC ACONTECEM QUANDO O INDIVÍDUO ESTÁ SE SENTINDO: [assinale uma ou mais opções]

( ) Ansioso
( ) Deprimido
( ) Impotente
( ) Culpado
( ) Nenhuma das anteriores

H) <u>COMPORTAMENTOS</u> DE PACIENTES COM TOC OS LEVAM A: [assinale uma ou mais opções]

( ) Precisarem de quantidades maiores do comportamento para alcançarem o efeito desejado
( ) Ficarem inquietos ou irritados quando tentam reduzir ou interromper o comportamento
( ) Tentativas malsucedidas de controlar, reduzir ou interromper o comportamento
( ) Ficarem preocupados com o comportamento
( ) Realizarem o comportamento quando se sentem angustiados (ex.: sentimentos de impotência, culpa, ansiedade, depressão)
( ) Repetirem o comportamento para ficarem quites ("recuperarem o prejuízo")
( ) Mentirem para esconder a extensão do envolvimento com o comportamento
( ) Prejudicarem ou perderem um relacionamento significativo, o emprego ou uma oportunidade educacional ou profissional por causa do comportamento
( ) Dependerem de outras pessoas por causa do comportamento
( ) Nenhuma das anteriores

Vá para a <u>Seção 4</u>.

SEÇÃO 4

- Para responder às próximas questões, por favor refira-se APENAS a ***experiências mentais*** (ex.: cognições)

EXPERIÊNCIAS MENTAIS **DE PACIENTES COM TOC INCLUEM:** [assinale uma ou mais opções]

( ) Pensamentos
( ) Impulsos
( ) Imagens
( ) Preocupações
( ) Fascinações ou atrações
( ) Interesses ou curiosidades
( ) Desejos ou fissuras
( ) Necessidades
( ) Sentimentos
( ) Medos
( ) Outra experiência mental
( ) Não são experiências mentais

Se você escolheu uma ou mais experiências mentais, vá para a letra A.
Se você escolheu a opção "Não são experiências mentais", pare de responder ao questionário.

A) EU ACHO QUE EXPERIÊNCIAS MENTAIS DE PACIENTES COM TOC: [assinale uma ou mais opções]

I. ( ) São claramente excessivas
II. ( ) Estão associadas à falta de controle
III. ( ) Estão associadas a tentativas repetidas e sem sucesso de:

a. ( ) Reduzi-las
b. ( ) Pará-las
c. ( ) Ignorá-las
d. ( ) Suprimi-las
e. ( ) Neutralizá-las
f. ( ) Resisti-las

IV. ( ) São projetadas para neutralizar ou prevenir outro fenômeno:

a. ( ) ***que está*** realisticamente conectado com elas (experiências mentais de pacientes com TOC estão logicamente relacionadas ao que elas visam neutralizar/prevenir)
b. ( ) ***que não está*** realisticamente conectado com elas (experiências mentais de pacientes com TOC não estão logicamente relacionadas ao que elas visam neutralizar/prevenir)

V. ( ) Nenhuma das anteriores

Continue respondendo às questões seguintes.

B) EXPERIÊNCIAS MENTAIS DE PACIENTES COM TOC ACONTECEM APESAR DE: [assinale uma ou mais opções]

( ) Problemas sociais ou interpessoais
( ) Riscos à integridade física
( ) Problemas psicológicos
( ) Nenhuma das anteriores

C) ALGUMAS DAS EXPERIÊNCIAS MENTAIS DE PACIENTES COM TOC LEVAM A: [assinale uma ou mais opções]

( ) Ansiedade ou sofrimento
( ) Prazer ou gratificação
( ) Alívio
( ) Nenhuma das anteriores

D) ENQUANTO OUTRAS TÊM O OBJETIVO DE: [assinale uma ou mais opções]

( ) Prevenir ansiedade ou sofrimento
( ) Reduzir ansiedade ou sofrimento
( ) Prevenir algum evento ou situação temida
( ) Oferecer prazer ou gratificação
( ) Oferecer alívio
( ) Nenhuma das anteriores

E) EXPERIÊNCIAS MENTAIS DE PACIENTES COM TOC SÃO PRECEDIDAS POR: [assinale uma ou mais opções]

( ) Tensão
( ) Excitação
( ) Nenhuma das anteriores

F) EXPERIÊNCIAS MENTAIS DE PACIENTES COM TOC ACONTECEM QUANDO O INDIVÍDUO ESTÁ SE SENTINDO: [assinale uma ou mais opções]

( ) Ansioso
( ) Deprimido
( ) Impotente
( ) Culpado
( ) Nenhuma das anteriores

G) PACIENTES COM TOC TAMBÉM: [assinale uma ou mais opções]

( ) Precisam de maiores quantidades da experiência mental para se sentirem satisfeitos
( ) Ficam inquietos ou irritados quando tentam não ter a experiência mental
( ) Sentem-se incapazes de controlar, reduzir ou interromper a experiência mental
( ) Ficam preocupados com a experiência mental
( ) Têm a experiência quando sentem-se angustiados (ex.: sentimentos de impotência, culpa, ansiedade, depressão)
( ) Mentem para esconder a experiência mental
( ) Prejudicam ou perdem um relacionamento significativo, o emprego ou uma oportunidade educacional ou profissional por causa da experiência mental
( ) Dependem de outras pessoas por causa da experiência mental
( ) Nenhuma das anteriores

PATIENT QUESTIONNAIRE

Identification: ________________________________ **Date: ___/___/____**

VALIDITY OF DSM-5 LINGUISTIC DESCRIPTIONS (PQ-VDLD)

Thinking about your main problem, answer the following questions according to your own opinion.

SECTION 1

MY MAIN PROBLEM CAN BE DEFINED AS: [choose one or more options]

( ) Recurrent
( ) Persistent
( ) Intrusive
( ) Unwanted
( ) Repetitive
( ) Excessive
( ) Inappropriate
( ) Compensatory
( ) Continued
( ) Unpleasant
( ) Habitual
( ) Unproductive
( ) Inflexible
( ) None of the above

SECTION 2

MY MAIN PROBLEM INCLUDES AN ATTEMPT TO AVOID OR RESTRICT: [choose one or more options]

( ) Contaminated objects
( ) Risky situations
( ) Sexual or blasphemous themes
( ) Asymmetric environments
( ) Mirror or reflective surfaces
( ) Discarding possessions or objects
( ) Energy intake or food
( ) Social situations
( ) Public transportation
( ) Open spaces
( ) Enclosed spaces
( ) Standing in line
( ) Crowds
( ) Outside home
( ) Other objects or situations
( ) It is not an attempt to avoid or restrict anything

SECTION 3

MY MAIN PROBLEM INCLUDES A BEHAVIOR, SUCH AS: [choose one or more options]

( ) Hand washing
( ) Ordering
( ) Checking
( ) Mirror checking
( ) Excessive grooming
( ) Not discarding possessions
( ) Acquisition
( ) Skin picking
( ) Reassurance seeking
( ) Pulling out of one's hair
( ) Eating (Energy intake)
( ) Outbursts (aggressive impulses)
( ) Fire setting
( ) Stealing
( ) Using alcohol
( ) Using drugs
( ) Gambling
( ) Praying
( ) Counting
( ) Repeating words silently
( ) Comparing appearance with that of others
( ) Other behavior
( ) It is not a behavior

If you chose one or more behaviors, go to letter A.
If you chose the option "It is not a behavior", go to Section 4.

A) MY BEHAVIORS ARE: [choose one or more options]

I. ( ) Clearly excessive
II. ( ) Associated with lack of control
III. ( ) Associated with repeated and unsuccessful attempts to be:

  a. ( ) Decreased or cut down
  b. ( ) Stopped
  c. ( ) Ignored
  d. ( ) Suppressed
  e. ( ) Neutralized
  f. ( ) Resisted

IV. ( ) Designed to neutralize or prevent other phenomena:

  a. ( ) that they ***are*** realistically connected with (i.e. my behaviors are logically related to what they aim to neutralize/prevent)
  b. ( ) that they ***are not*** realistically connected with (i.e. my behaviors are not logically related to what they aim to neutralize/prevent)

V. ( ) None of the above

Now keep answering the following questions.

B) MY BEHAVIORS ARE PERFORMED DESPITE: [choose one or more options]

( ) Social or interpersonal problems
( ) Physical hazards
( ) Psychological problems
( ) None of the above

C) SOME OF MY BEHAVIORS LEAD TO: [choose one or more options]

( ) Anxiety or distress
( ) Pleasure or gratification
( ) Relief
( ) None of the above

D) WHILE OTHERS ARE AIMED AT: [choose one or more options]

( ) Preventing anxiety or distress
( ) Reducing anxiety or distress
( ) Preventing some dreaded event or situation
( ) Obtaining pleasure or gratification
( ) Obtaining relief
( ) None of the above

E) MY BEHAVIORS ARE PERFORMED IN RESPONSE OR ACCORDING TO: [choose one or more options]

( ) Thoughts
( ) Urges
( ) Images
( ) Preoccupations
( ) Fascinations or attractions
( ) Interests or curiosities
( ) Desires or cravings
( ) Needs
( ) Feelings
( ) Fears
( ) Appearance concerns
( ) Rigid rules
( ) A force
( ) An obligation
( ) A pressure
( ) None of the above

F) MY BEHAVIORS ARE PRECEEDED BY: [choose one or more options]

( ) Tension
( ) Affective arousal
( ) None of the above

G) MY BEHAVIORS OCCUR WHEN I AM FEELING: [choose one or more options]

( ) Anxious
( ) Depressed
( ) Helpless
( ) Guilty
( ) None of the above

H) REGARDING YOUR PROBLEMATIC BEHAVIOR, CHOOSE ONE OR MORE OPTIONS:

( ) I need increased amounts of behavior to achieve the desired effect
( ) I become restless or irritable when attempting to cut down or stop performing behavior
( ) I unsuccessfully attempt to control, cutback or stop the behavior
( ) I become preoccupied with the behavior
( ) I perform the behavior when feeling distressed (e.g. helpless, guilty, anxious, depressed)
( ) I return to get things even
( ) I lie to conceal the extent of involvement with behavior
( ) I jeopardize or lose a significant relationship, job, or educational career opportunity because of behavior
( ) I rely on others because of behavior
( ) None of the above

Go to Section 4.

SECTION 4

MY MAIN PROBLEM INCLUDES A MENTAL EXPERIENCE, SUCH AS: [choose one or more options]

( ) Thoughts
( ) Urges
( ) Images
( ) Preoccupations
( ) Fascinations or attractions
( ) Interests or curiosities
( ) Desires or cravings
( ) Needs
( ) Feelings
( ) Fears
( ) Other mental experience
( ) It is not a mental experience

If you chose one or more experiences, go to letter A.
If you chose the option "It is not a mental experience", stop answering this questionnaire.

A) MY MENTAL EXPERIENCES ARE: [choose one or more options]

I.) ( ) Clearly excessive
II.) ( ) Associated with lack of control
III.) ( ) Associated with repeated and unsuccessful attempts to be:

a. ( ) Decreased or cut down
b. ( ) Stopped
c. ( ) Ignored
d. ( ) Suppressed
e. ( ) Neutralized
f. ( ) Resisted

IV.) ( ) Designed to neutralize or prevent other phenomena:

a. ( ) that they ***are*** realistically connected with (i.e. my mental experiences are logically related to what they aim to neutralize/prevent)
b. ( ) that they ***are not*** realistically connected with (i.e. my mental experiences are not logically related to what they aim to neutralize/prevent)

Now keep answering the following questions.

B) MY MENTAL EXPERIENCES HAPPEN DESPITE: [choose one or more options]

( ) Social or interpersonal problems
( ) Physical hazards
( ) Psychological problems
( ) None of the above

C) SOME OF MY MENTAL EXPERIENCES LEAD TO: [choose one or more options]

( ) Anxiety or distress
( ) Pleasure or gratification
( ) Relief
( ) None of the above

D) WHILE OTHERS ARE AIMED AT: [choose one or more options]

( ) Prevent anxiety or distress
( ) Reduce anxiety or distress
( ) Prevent some dreaded event or situation
( ) Get me pleasure or gratification
( ) Get me relief
( ) None of the above

E) MY MENTAL EXPERIENCES ARE PRECEEDED BY: [choose one or more options]

( ) Tension
( ) Affective arousal
( ) None of the above

F) MY MENTAL EXPERIENCES OCCUR WHEN I AM FEELING: [choose one or more options]

( ) Anxious
( ) Depressed
( ) Helpless
( ) Guilty
( ) None of the above

G) REGARDING YOUR PROBLEMATIC MENTAL EXPERIENCES, CHOOSE ONE OR MORE OPTIONS:

( ) I need increased amounts of the mental experience to feel satisfied
( ) I become restless or irritable when attempting to not have the mental experience
( ) I feel unable to control, cutback or stop the mental experience
( ) I become preoccupied with the mental experience
( ) I experience it when feeling distressed (e.g. helpless, guilty, anxious, depressed)
( ) I lie to conceal the mental experience
( ) I jeopardize or lose a significant relationship, job, or educational career opportunity because of the mental experience
( ) I rely on others because of the mental experience
( ) None of the above

QUESTIONÁRIO DO PACIENTE

Identificação: ______________________________ **Data:** ___/___/_____

VALIDAÇÃO DAS DESCRIÇÕES LINGUÍSTICAS DO DSM-5 (QP-VDLD)

Pensando sobre o seu maior problema, responda às seguintes questões de acordo com sua opinião pessoal.

SEÇÃO 1

MEU PROBLEMA PODE SER DEFINIDO COMO: [escolha uma ou mais opções]

( ) Recorrente
( ) Persistente
( ) Intrusivo
( ) Indesejado
( ) Repetitivo
( ) Excessivo
( ) Inapropriado
( ) Compensatório
( ) Contínuo
( ) Desagradável
( ) Habitual
( ) Improdutivo
( ) Inflexível
( ) Nenhuma das anteriores

SEÇÃO 2

MEU PROBLEMA PRINCIPAL INCLUI UMA TENTATIVA DE EVITAR OU RESTRINGIR: [escolha uma ou mais opções]

( ) Objetos contaminados
( ) Situações de risco
( ) Blasfêmias ou temas sexuais
( ) Ambientes assimétricos
( ) Espelhos ou superfícies refletoras
( ) Descartar pertences ou objetos
( ) Ingestão calórica ou alimentos
( ) Situações sociais
( ) Transporte público
( ) Espaços abertos
( ) Locais fechados
( ) Ficar em uma fila
( ) Multidões
( ) Sair de casa
( ) Outros objetos ou situações
( ) Não é uma tentativa de evitar ou restringir coisas

SEÇÃO 3

MEU PROBLEMA PRINCIPAL INCLUI UM COMPORTAMENTO, COMO: [escolha uma ou mais opções]

( ) Lavagem de mãos
( ) Ordenamento
( ) Verificação/checagem
( ) Verificação no espelho
( ) Arrumação excessiva
( ) Não descartar pertences
( ) Aquisição
( ) Beliscar a pele
( ) Buscar tranquilização
( ) Arrancar os próprios cabelos
( ) Comer (ingestão calórica)
( ) Explosividade (impulsos agressivos)
( ) Provocar incêndios
( ) Roubar
( ) Uso de álcool
( ) Uso de drogas
( ) Apostas
( ) Rezas
( ) Contagem
( ) Repetição de palavras em silêncio
( ) Comparação da aparência com a de outros
( ) Outro comportamento
( ) Meu problema não é um comportamento

Se você escolheu um ou mais comportamentos, vá para a letra A.
Se você escolheu a opção "Meu problema não é um comportamento", vá para a Seção 4.

A) MEUS COMPORTAMENTOS: [escolha uma ou mais opções]

I. ( ) São claramente excessivos
II. ( ) Estão associados com falta de controle
III. ( ) Estão associados com tentativas repetidas e sem sucesso de:

   a. ( ) Reduzi-los
   b. ( ) Pará-los
   c. ( ) Ignorá-los
   d. ( ) Suprimi-los
   e. ( ) Neutralizá-los
   f. ( ) Resisti-los

IV. ( ) São projetados para neutralizar ou prevenir outro fenômeno:

   a. ( ) ***que está*** realisticamente conectado com eles (meus comportamentos estão logicamente relacionados ao que eles visam neutralizar/prevenir)
   b. ( ) ***que não está*** realisticamente conectado com eles (meus comportamentos não estão logicamente relacionados ao que eles visam neutralizar/prevenir)

V. ( ) Nenhuma das anteriores

Continue respondendo às questões seguintes.

B) MEUS COMPORTAMENTOS CONTINUAM, APESAR DE: [escolha uma ou mais opções]

( ) Problemas sociais ou interpessoais
( ) Riscos à integridade física
( ) Problemas psicológicos
( ) Nenhuma das anteriores

C) ALGUNS DOS MEUS COMPORTAMENTOS LEVAM A: [escolha uma ou mais opções]

( ) Ansiedade ou sofrimento
( ) Prazer ou gratificação
( ) Alívio
( ) Nenhuma das anteriores

D) ENQUANTO OUTROS TÊM O OBJETIVO DE: [escolha uma ou mais opções]

( ) Prevenir ansiedade ou sofrimento
( ) Reduzir ansiedade ou sofrimento
( ) Prevenir algum evento ou situação temida
( ) Obter prazer ou gratificação
( ) Obter alívio
( ) Nenhuma das anteriores

E) MEUS COMPORTAMENTOS SÃO REALIZADOS EM RESPOSTA OU DE ACORDO COM: [escolha uma ou mais opções]

( ) Pensamentos
( ) Impulsos
( ) Imagens
( ) Preocupações
( ) Fascinações ou atrações
( ) Interesses ou curiosidades
( ) Desejos ou fissuras
( ) Necessidades
( ) Sentimentos
( ) Medos
( ) Preocupações com a aparência
( ) Regras rígidas
( ) Uma força
( ) Uma obrigação
( ) Uma pressão
( ) Nenhuma das anteriores

F) MEUS COMPORTAMENTOS SÃO PRECEDIDOS POR: [escolha uma ou mais opções]

( ) Tensão
( ) Excitação
( ) Nenhuma das anteriores

G) MEUS COMPORTAMENTOS ACONTECEM QUANDO ESTOU ME SENTINDO:
[escolha uma ou mais opções]

( ) Ansioso(a)
( ) Deprimido(a)
( ) Impotente
( ) Culpado(a)
( ) Nenhuma das anteriores

H) EM RELAÇÃO AO SEU COMPORTAMENTO PROBLEMÁTICO, ESCOLHA UMA OU MAIS OPÇÕES:

( ) Eu preciso de quantidades maiores do comportamento para alcançar o efeito desejado
( ) Eu fico inquieto ou irritado quando tento reduzir ou interromper o comportamento
( ) Minhas tentativas de controlar, reduzir ou interromper o comportamento são malsucedidas
( ) Eu fico preocupado(a) com o comportamento
( ) Eu realizo o comportamento quando me sinto angustiado(a) (ex.: sentimentos de impotência, culpa, ansiedade, depressão)
( ) Eu repito o comportamento para ficar quite ("recuperar o prejuízo")
( ) Eu minto para esconder a extensão do meu envolvimento com o comportamento
( ) Eu prejudico ou perco um relacionamento significativo, o emprego ou uma oportunidade educacional ou profissional por causa do comportamento
( ) Eu dependo de outras pessoas por causa do comportamento
( ) Nenhuma das anteriores

Vá para a Seção 4.

SEÇÃO 4

MEU PROBLEMA PRINCIPAL INCLUI UMA EXPERIÊNCIA MENTAL, COMO:
[escolha uma ou mais opções]

( ) Pensamentos
( ) Impulsos
( ) Imagens
( ) Preocupações
( ) Fascinações ou atrações
( ) Interesses ou curiosidades
( ) Desejos ou fissuras
( ) Necessidades
( ) Sentimentos
( ) Medos
( ) Outra experiência mental
( ) Meu problema não é uma experiência mental

Se você escolheu um ou mais comportamentos, vá para a letra A.
Se você escolheu a opção "Meu problema não é uma experiência mental", pare de responder ao questionário.

A) MINHAS EXPERIÊNCIAS MENTAIS: [escolha uma ou mais opções]

I.) ( ) São claramente excessivas
II.) ( ) Estão associadas com falta de controle
III.) ( ) Estão associadas com tentativas repetidas e sem sucesso de:

a. ( ) Reduzi-las
b. ( ) Pará-las
c. ( ) Ignorá-las
d. ( ) Suprimi-las
e. ( ) Neutralizá-las
f. ( ) Resisti-las

IV.) ( ) São projetadas para neutralizar ou prevenir outro fenômeno:

a. ( ) ***que está*** realisticamente conectado com elas (minhas experiências mentais estão logicamente relacionadas ao que elas visam neutralizar/prevenir)
b. ( ) ***que não está*** realisticamente conectado com elas (minhas experiências mentais não estão logicamente relacionadas ao que elas visam neutralizar/prevenir)

Continue respondendo às questões seguintes.

B) MINHAS EXPERIÊNCIAS MENTAIS CONTINUAM, APESAR DE: [escolha uma ou mais opções]

( ) Problemas sociais ou interpessoais
( ) Riscos à integridade física
( ) Problemas psicológicos
( ) Nenhuma das anteriores

C) ALGUMAS DAS MINHAS EXPERIÊNCIAS MENTAIS ME LEVAM A: [escolha uma ou mais opções]

( ) Ansiedade ou sofrimento
( ) Prazer ou gratificação
( ) Alívio
( ) Nenhuma das anteriores

D) ENQUANTO OUTRAS TEM O OBJETIVO DE: [escolha uma ou mais opções]

( ) Prevenir ansiedade ou sofrimento
( ) Reduzir ansiedade ou sofrimento
( ) Prevenir algum evento ou situação temida
( ) Obter prazer ou gratificação
( ) Obter alívio
( ) Nenhuma das anteriores

E) MINHAS EXPERIÊNCIAS MENTAIS SÃO PRECEDIDAS POR: [escolha uma ou mais opções]

( ) Tensão
( ) Excitação
( ) Nenhuma das anteriores

F) MINHAS EXPERIÊNCIAS MENTAIS ACONTECEM QUANDO ESTOU ME SENTINDO: [escolha uma ou mais opções]

( ) Ansioso(a)
( ) Deprimido(a)
( ) Impotente
( ) Culpado(a)
( ) Nenhuma das anteriores

G) EM RELAÇÃO AS SUAS EXPERIÊNCIAS MENTAIS PROBLEMÁTICAS, ESCOLHA UMA OU MAIS OPÇÕES:

( ) Eu preciso de quantidades maiores da experiência mental para me sentir satisfeito(a)
( ) Eu fico inquieto(a) ou irritado(a) quando tento não ter a experiência mental
( ) Eu me sinto incapaz de controlar, reduzir ou interromper a experiência mental
( ) Eu fico preocupado(a) com a experiência mental
( ) Eu tenho a experiência mental quando me sinto angustiado(a) (ex.: sentimentos de impotência, culpa, ansiedade, depressão)
( ) Eu minto para esconder a experiência mental
( ) Eu prejudico ou perco um relacionamento significativo, o emprego ou uma oportunidade educacional ou profissional por causa da experiência mental
( ) Eu dependo de outras pessoas por causa da experiência mental
( ) Nenhuma das anteriores
