# Supplementary material for: Do patients and clinicians agree on the essential features of OCD?
Source: CNS Spectr. 2026 Jun 17;31(1):e21. doi: 10.1017/S1092852926101011 (PMC13732608; doi:10.1017/S1092852926101011)
Supplement: Supplementary file 2 [file S1092852926101011sup002.docx]

**Table S1. Patient–clinician comparison of DSM-5–derived descriptors based on clinicians’ “essential to diagnosis” responses**

|  |  | DSM-5 Chapter Source |  | Expert clinicians | |  | OCD patients | |  | Statistics |  | General Findings |
| --- | --- | --- | --- | --- | --- | --- | --- | --- | --- | --- | --- | --- |
|  |  |  |  | [N_essential_ (%)] | [N_missing_ (%)] |  | [N_essential_ (%)] | [N_missing_ (%)] |  |  |  |  |
| Recurrent |  | **OCD**, TR, SP, BN, BED, IED, KM, AUD, CUD, PUD, OHD, IUD, OUD, SHA, SUD, TUD, OSD, GD |  | 29 (80.6) | 0 (0.0) |  | 27 (42.2) | 0 (0.0) |  | p < 0.001* |  | Clinician > Patient |
| Persistent |  | **OCD**, HD, AN, CD, AUD, CUD, PUD, OHD, IUD, OUD, SHA, SUD, TUD, OSD, GD |  | 28 (77.8) | 0 (0.0) |  | 36 (56.3) | 0 (0.0) |  | p = 0.031* |  | Clinician > Patient |
| Intrusive |  | **OCD**, BDD |  | 34 (94.4) | 0 (0.0) |  | 32 (50.0) | 0 (0.0) |  | p < 0.001* |  | Clinician > Patient |
| Unwanted |  | **OCD**, BDD |  | 31 (86.1) | 0 (0.0) |  | 49 (76.6) | 0 (0.0) |  | p = 0.252 |  | Clinician = Patient |
| Repetitive |  | **OCD**, BDD, CD |  | 32 (88.9) | 0 (0.0) |  | 46 (71.9) | 0 (0.0) |  | p = 0.049* |  | Clinician > Patient |
| Excessive |  | **OCD**, BDD, HD, BN, BED |  | 25 (71.4) | 1 (2.8) |  | 24 (37.5) | 0 (0.0) |  | p = 0.001* |  | Clinician > Patient |
| Inappropriate |  | BN |  | 16 (44.4) | 0 (0.0) |  | 23 (35.9) | 0 (0.0) |  | p = 0.402 |  | Clinician = Patient |
| Continued |  | AUD, CUD, PUD, OHD, IUD, OUD, SHA, SUD, TUD, OSD |  | 5 (14.3) | 1 (2.8) |  | 24 (37.5) | 0 (0.0) |  | p = 0.015* |  | Clinician < Patient |
| Unpleasant |  | AUD |  | 17 (48.6) | 1 (2.8) |  | 48 (75.0) | 0 (0.0) |  | p = 0.008* |  | Clinician < Patient |
| Habitual |  | AUD |  | 5 (14.3) | 1 (2.8) |  | 18 (28.1) | 0 (0.0) |  | p = 0.119 |  | Clinician = Patient |
| Inflexible |  | AN |  | 9 (25.7) | 1 (2.8) |  | 8 (12.5) | 0 (0.0) |  | p = 0.096 |  | Clinician = Patient |
| Unproductive |  | - |  | 13 (36.1) | 0 (0.0) |  | 22 (34.4) | 0 (0.0) |  | p = 0.861 |  | Clinician = Patient |
| Compensatory |  | BN |  | 6 (17.1) | 1 (2.8) |  | 7 (10.9) | 0 (0.0) |  | p = 0.382 |  | Clinician = Patient |

Footnote: OCD = obsessive-compulsive disorder; BDD = body dysmorphic disorder; HD = hoarding disorder; TR = trichotillomania; SP = skin-picking; AN = anorexia nervosa; BN = bulimia nervosa; BED = binge-eating disorder; IED = intermittent explosive disorder; CD = conduct disorder; PM = pyromania; KM = kleptomania; AUD = alcohol use disorder; CUD = cannabis use disorder; PUD = phencyclidine use disorder; OHD = other hallucinogen use disorder; IUD = inhalant use disorder; OUD = opioid use disorder; SHA = sedative, hypnotic, or anxiolytic use disorder; SUD = stimulant use disorder; TUD = tobacco use disorder; OSD = other (or unknown) substance use disorder; GD = gambling disorder; * = p < 0.05
